# Definitions

**Published:** 1883-09

**Authors:** 


### DEFINITIONS.

Wrinkles—Life's scars.

Tears—Soul's blood.

Baldness—Work's crown and vice's reward.

Chocolate—A nutritious paste, in whose composition enters a little of everything, even Cocoa.—*El Siglo Medico*, Madrid.

